# Targeted management of evolving and established chronic lung disease of prematurity assisted by cardiopulmonary ultrasound: A case report of four patients

**DOI:** 10.3389/fped.2022.1112313

**Published:** 2023-01-30

**Authors:** Guglielmo Bruno, Roberto Chioma, Enrico Storti, Giovanni De Luca, Margherita Fantinato, Patrizio Antonazzo, Maria Pierro

**Affiliations:** ^1^ Neonatal and Paediatric Intensive Care Unit, M. Bufalini Hospital, AUSL Romagna, Cesena, Italy; ^2^ Paediatric Unit, Azienda Ospedaliero-Universitaria di Ferrara, Ferrara, Italy; ^3^ Dipartimento Universitario Scienze Della Vita e Sanità Pubblica, Unità Operativa Complessa di Neonatologia, Fondazione Policlinico Universitario A. Gemelli Istituto di Ricovero e Cura a Carattere Scientifico, Università Cattolica del Sacro Cuore, Rome, Italy; ^4^ Department of Critical Care, Maggiore Hospital, Cremona, Cremona, Italy; ^5^ Pathologic Anatomy Oncohematology Department, M. Bufalini Hospital, AUSL Romagna, Cesena, Italy; ^6^ Unit of Obstetrics and Gynecology, Bufalini Hospital-AUSL Romagna, Cesena, Italy

**Keywords:** lung ultrasonography (LUS), targeted neonatal echocardiography, phenotype (mesh), endotype, bronchopulmonary dysplasia (BPD), case report

## Abstract

Bronchopulmonary dysplasia (BPD) is one of the most common complications of premature birth. The current definition of BPD is based on the duration of oxygen therapy and/or respiratory support. Among the pitfalls of all the diagnostic definitions, the lack of a proper pathophysiologic classification makes it difficult to choose an appropriate drug strategy for BPD. In this case report, we describe the clinical course of four premature infants, admitted to the neonatal intensive care unit, for whom the use of lung and cardiac ultrasound was an integral part of the diagnostic and therapeutic process. We describe, for the first time to our knowledge, four different cardiopulmonary ultrasound patterns of evolving and established chronic lung disease of prematurity and the consequent therapeutic choices. This approach, if confirmed in prospective studies, may guide the personalized management of infants suffering from evolving and established BPD, optimizing the chances of success of the therapies and at the same time reducing the risk of exposure to inadequate and potentially harmful drugs.

## Introduction

Bronchopulmonary dysplasia (BPD), also known as chronic lung disease (CLD) of prematurity, is one of the most common, serious complications of premature birth (1–3). BPD definition has changed over the years and still has several pitfalls. The major problem with all the BPD definitions is that the term BPD encompasses various forms of injury that may differently affect lung compartments. Airways, alveoli, vessels, interstitium, and lymphatic system may be altered in a relatively specific way, giving rise to different clinical phenotypes of BPD (4). These physiopathological insights have not yet been included in the BPD definition, as the different phenotypes are difficult to unveil in clinical practice.

Lung Ultrasound (LUS) is being increasingly used as a diagnostic tool in Neonatal Intensive Care Units (NICUs). LUS is used to diagnose several respiratory diseases (5), adjust mechanical ventilation (6, 7), and predict BPD (8). Targeted neonatal echocardiography (TnEcho) is commonly performed to assess cardiac function and evaluate the need for cardiovascular interventions in preterm and term neonates (9). However, the combination of TnEcho and LUS to identify different sonographic patterns of lung disease and guide treatment with an integrated Point of Care Ultrasound (POCUS) strategy remains largely unexplored. In this report of four cases, we describe how integrated POCUS guided a therapeutic approach based on pathophysiological assumptions and sonographic findings, monitoring the response to interventions. LUS focused on type and distribution of B-Lines, which are long, vertical, hyperechoic, dynamic lines originating from the pleural line and suggesting a plural-interstitial lung disease. TnEcho examination checked for the presence of hemodynamically significant patent ductus arteriosus (hsPDA) and indirect signs of pulmonary hypertension, including a shorter pulmonary artery acceleration time/ejection time ratio (AT/ET) and an increased left ventricle eccentricity index (EI) (10). A decreasing trend in the plasmatic N-terminal pro-b-type natriuretic peptide (NT-proBNP) values was used to assess response to therapy in the case of pulmonary hypertension (PH) (11).

Furthermore, we report the placental histology analysis, linking the endotypes of prematurity (infection/inflammation and dysfunctional placentation) and the subsequent development of specific pathways of lung injury, leading to sonographic cardio-pulmonary phenotypes.

## Case description

### Patient 1

**Figure 1:**
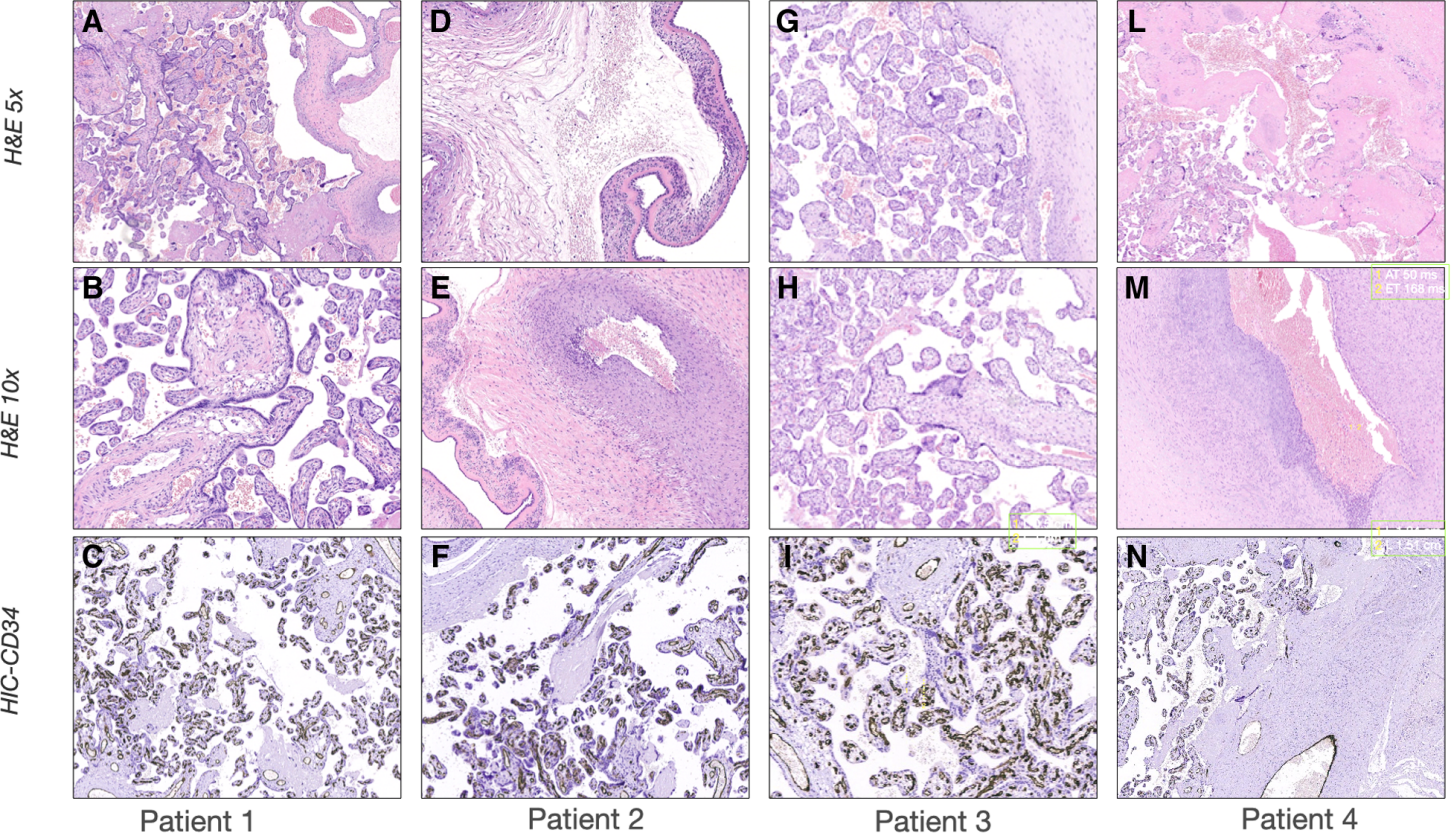
Sonographic patterns of bronchodysplasia (BPD). Representative pictures of Lung Ultrasound (LUS) and echocardiography of the patients at the time of the beginning of therapy. (**A–C**) *Case 1:* (**A**) LUS showing homogenously and gravity-related distributed B-lines with a normal pleural line; (**B**) color-Doppler of the pulmonary artery showing normal flow; (**C**) B-mode evaluation of ventricular chambers showing normal eccentricity index (EI). (**D-F**) *Case 2*: (**D**) LUS showing inhomogeneous B-line distribution with spared areas of normal sonographic lung appearance, surrounded by areas containing multiple B-lines. Pleural line thickened and irregular; (**E**) color-Doppler of the pulmonary artery showing normal flow; (**F**) B-mode evaluation of ventricular chambers showing normal EI. (**G-I**) *Case 3*: (**G**) LUS homogenously and gravity-related distributed B-lines with subpleural atelectasis of the dependent regions; (**H**) color-Doppler of the patent ductus arteriosus showing pulsatile flow; (**I**) M-mode evaluation of the left atrium (LA) to aorta (Ao) ratio, showing important dilation of the ratio atrium (LA:Ao = 2); (**L-N**) *Case 4*: (**L**) LUS showing confluent B-lines with sparing areas, thickened pleural line, and with subpleural atelectasis; (**M**) color-Doppler of the pulmonary artery showing decreased acceleration time/ejection time (AT/ET) = 0.28; (**N**) B-mode evaluation of ventricular chambers showing decreased EI (EI = 1.2).

**Figure 2:**
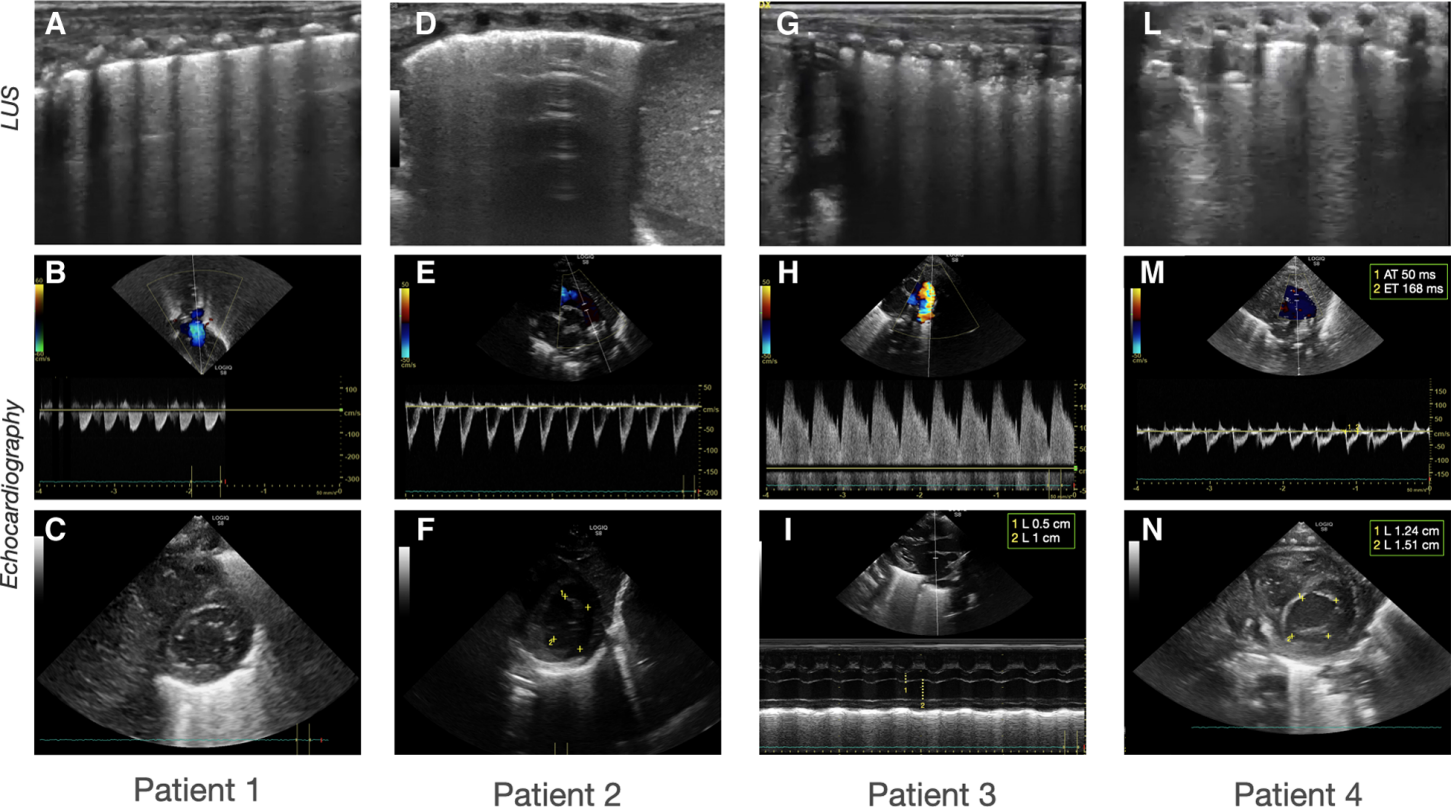
Histological examination of the placentas. Representative pictures of the histological examination of the placenta. H & E 5×: Hematoxylin and eosin stain at 5 magnifications; H & E 10×: Hematoxylin and eosin stain at 10 magnification; IHC-CD34: Immunohistochemistry stains for cluster of differentiation. (**A-C**) *Case 1:* (**A**) H & E showing normal placenta for the gestational age with mild signs of chorioamnionitis; (**B**) H & E showing absence of vasculitis; (**C**) IHC showing normal distribution of CD34 in the placental vessels. (**D-F**) *Case 2*: (**D**) H & E stain showing severe chorioamnionitis; (**E**) H & E stain showing severe vasculitis; (**F**) IHC showing normal distribution of CD34 in the placental vessels. (**G-I**) *Case 3*: (**G**) H & E showing normal placenta for the gestational age with mild signs of chorioamnionitis; (**H**) H & E showing absence of vasculitis; (**I**) IHC showing normal distribution of CD34 in the placental vessels; (**L-N**) *Case 4*: (**L**) H & E showing severe chorioamnionitis and acute ischemic area of malperfusion; (**M**) H & E stain showing severe vasculitis and funisitis; (**N**) IHC showing severe suppression of CD34 expression.

The first case was a male neonate born at 26 weeks' gestation by emergency cesarian section, due to the onset of preterm labor and cord prolapse of the second twin in a dichorionic-diamniotic twin pregnancy. The patient was intubated in the delivery room due to poor respiratory effort (Table 1). The patient subsequently developed respiratory distress syndrome (RDS), treated with two doses of exogenous surfactant. He was successfully extubated at 10 h of life, receiving continuous positive airway pressure (CPAP) on room air. The placental histology examination was unremarkable (Figure 1). The first week of life was complicated by *Enterococcus Faecium* late-onset sepsis (day of life 6). During the episode, the patient experienced a worsening of the general clinical and respiratory conditions, being switched to bilevel positive airway pressure (Bi-PAP) and requiring a fraction of inspired oxygen (FiO2) up to 0.35. The patient was treated with broad-spectrum antibiotics for 5 days, with normalization of the inflammatory markers and clinical conditions. However, the respiratory status did not improve. Hence, POCUS was performed at day 15 of life (28 + 1 weeks of post-menstrual age (PMA)). TnEcho was normal, while LUS revealed multiple B-lines on the posterior lung fields (Figure 2). As the assessment was performed with the patient in the supine position, he was switched to the prone position, aiming to repeat the assessment after two hours. At the following LUS examination, diffuse symmetrical and homogenous B-lines were observed in the anterior dependent lung fields, with normal A-pattern on the posterior areas. Since the respiratory status of the patient was deteriorating in terms of work of breathing and oxygen need, the LUS exam was repeated after two days, confirming the same pattern and showing further signs of lung fluid overload. Given the homogenous and gravity-dependent B-line distribution, the pattern was interpreted as idiopathic evolving pulmonary edema without any secondary cause (3). Therefore, a 7-day course of loop diuretics was commenced. The infant responded promptly to the therapy, being switched from Bi-PAP to CPAP in one day and weaned from oxygen in three. LUS picture normalized as well in all lung fields after treatment. Starting from the day 22 of life (29 + 1 weeks of PMA) he received non-invasive respiratory support with high-flow nasal cannula (HFNC) for 43 days (35 + 3 weeks of PMA). The patient did not meet the recently published criteria for BPD at 36 weeks of PMA (12) and was discharged home in good conditions.

**Table 1:** Clinical characteristics of the patients.

	Patient 1	Patient 2	Patient 3	Patient 4
GA (weeks)	26 + 0	26 + 0	28 + 0	30 + 2
BW (grams)	850	945	835	1730
Sex	Male	Male	Male	Male
Delivery	Cesarean section	Cesarean section	Cesarean section	Vaginal delivery
Pregnancy complications	Cervical insufficiency Twin pregnancy	Cervical insufficiency Twin pregnancy Umbilical cord prolapse	pPROM Twin pregnancy IUGR	pPROM (from GA 26 + 3 w) Anhydramnios
Maternal steroid	2 doses of betamethasone 2 days before delivery	2 doses of betamethasone 2 days before delivery	2 doses of betamethasone 16 days before delivery	2 doses of betamethasone 30 days before delivery
Delivery resuscitation	PPV, intubation and chest compression	PPV and nCPAP	PPV (intubation)	PPV (intubation)
Apgar scores	4–7	8–9	4–8	5–7
Surfactant therapy (number of doses)	2	1	0	1
Invasive MV duration (days)	1	3	6	4
CPAP duration (days)	19	15	34	4
HFNC duration (days)	43	38	19	7
Need for O2 (days)	33	68	46	11
Suspension of oxygen/respiratory dependency (weeks PMA)	35 + 3	35 + 5	36 + 1	37 + 6

Definition: GA gestational age, BW birth weight, MV mechanical ventilation, HFNC high flow nasal cannula, PPV positive pressure ventilation, pPROM preterm premature rupture of membrane, MV mechanical ventilation, IUGR intrauterine growth restriction.

### Patient 2

**Figure 3:**
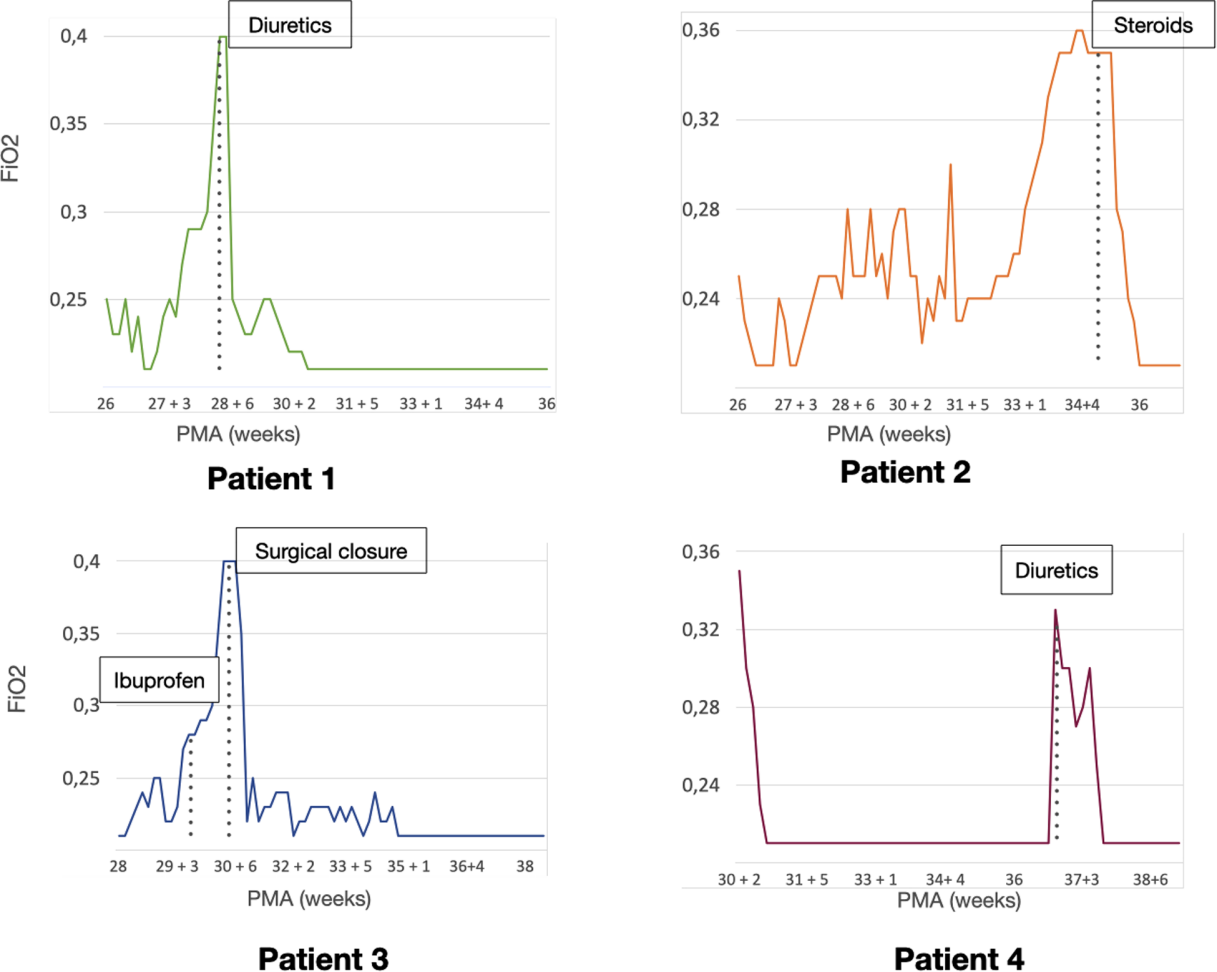
Patient clinical course. Graphics representing the need for respiratory assistance of each patient. The vertical axis shows the mean fraction of inspired oxygen (FiO2) needs for each day, the horizontal axis represents the post-menstrual age (PMA). The time-point for each specific (cardiopulmonary ultrasound (CPUS) guided intervention is highlighted in each graphic.

This male neonate was the twin brother of Patient 1. His delivery was complicated by umbilical cord prolapse. Being assisted with non-invasive ventilation (CPAP) in the delivery room and at NICU admission, the endotracheal surfactant administration due to RDS was performed *via* INtubation-SURfactant-Extubation (INSURE) technique at 4 h of life, with a reduction of the FiO2 from 0.55 to 0.25 (Table 1). The infant was successfully extubated to CPAP after the INSURE procedure. Placental histology examination revealed severe acute chorioamnionitis with vasculitis, in addition to a non-significant mild chronic underperfusion (Figure 1). Starting from 33 weeks PMA, oxygen need further increased and the patient required mechanical ventilation for 6 days between 33 + 2 and 34 + 0 weeks because of CPAP belly, respiratory insufficiency and intractable apnea, with no other apparent reasons (Figure 3). TnEcho was normal, but LUS showed multiple diffuse B-lines with irregular distribution in both anterior and posterior lung fields. Furthermore, pleural line appeared thickened, coarse, and irregular (Figure 2). To confirm the gravity-independent distribution of the B-lines, the patient was switched to prone position and the examination was repeated after two hours, revealing unchanged findings in the B-line distribution.

This POCUS pattern of lung injury was interpreted as an evolving interstitial lung disease (ILD), potentially triggered by ongoing inflammation (3). On the day of life 64 (35 + 1 weeks of PMA), a low-dose dexamethasone therapy was commenced, for a total duration of ten days. The oxygen was weaned in two days and the infant was switched to HFNC for four days after the initiation of the therapy. The infant was spontaneously breathing on room air starting from 36 PMA. The patient did not meet the recently published criteria for BPD diagnosis (12) and was discharged home in good conditions.

### Patient 3

This male neonate was born at 28 weeks of gestation by cesarean section due to non-reassuring fetal status after the onset of labor. The monochorionic-diamniotic twin pregnancy was complicated by preterm premature rupture of membranes (pPROM) and intrauterine growth restriction (IUGR). The patient was intubated in the delivery room due to poor respiratory effort and did not necessitate endotracheal surfactant administration (Table 1). He was successfully extubated at 4 h of life, receiving CPAP on room air. Placental histology was unremarkable (Figure 1). At 7 days of life, because of a large HsPDA, treatment with a five-day course of intravenous paracetamol was commenced. As the clinical response was poor and TnEcho showed the persistence of HsPDA, a course of intravenous ibuprofen was initiated at 12 days of life, without success, while the patient's oxygen need gradually increased (FiO2 0.35). A comprehensive LUS assessment was performed at 20 days of life (30 + 5 weeks of PMA), which showed confluent B-lines in the posterior lung fields with several subpleural atelectasis, and normal anterior lung patterns. Two hours after switching the patient to the prone position, LUS revealed the resolution of posterior atelectasis, while confluent B-lines appeared on the anterior lung fields; meanwhile, the oxygenation transiently improved, presumably due to the reopening of the posterior respiratory units. The findings were interpreted as pulmonary edema caused by pulmonary overcirculation due to HsPDA. The clinical and POCUS picture supported the choice of performing the surgical closure of the PDA (Figure 2). The infant's position was often modified according to the LUS findings to avoid the development of dependent atelectasis and loop diuretic treatment was started, until the day of surgical closure (22 days of life, 31 + 1 weeks of PMA). After the surgery, the respiratory conditions rapidly improved, and the infant was weaned from oxygen on the day of life 49 (35 weeks of PMA) and from non-invasive ventilation on the day of life 57 (36 + 1 weeks of PMA). The patient was diagnosed with grade I BPD (12) and was discharged home in good condition.

### Patient 4

The fourth case was a male infant born *via* vaginal delivery at 30.3 weeks of gestational age. The pregnancy was complicated by pPROM and anhydramios from 26 weeks of gestation. At birth, the patient was intubated due to poor respiratory drive. Chest x-ray and LUS were performed right after birth, resulting suggestive of severe RDS and lung hypoplasia (Table 1). Endotracheal surfactant instillation allowed a drastic improvement in oxygenation, decreasing FiO2 from 0.8 to 0.25. After four days of high-frequency oscillatory ventilation, the baby was extubated to CPAP. Oxygen and non-invasive ventilation were promptly weaned, and the patient was breathing without support on room air on the day of life 8 (31 + 4 weeks of PMA) (Figure 3). Histological examination of the placenta revealed severe acute chorioamnionitis interesting chorion and subchorionic parenchyma, culminating into severe funisitis. Furthermore, significant signs of acute and chronic placental underperfusion were reported (Figure 1). On the day of life 44 (36 + 5 weeks of PMA), the infant experienced several hypoxic episodes, not related to feeds and without increased work of breathing. The septic workup (C-reactive protein, complete blood count, procalcitonin) was negative. Low-flow oxygen (FiO2 0.40) was administrated to achieve adequate saturation. LUS revealed localized posterior atelectasis, while TnEcho showed signs of pulmonary hypertension (PH) (Figure 2). To recruit the collapsed fields and optimize pulmonary hemodynamics, the patient was placed in the prone position, employing LUS to monitor the reopening of atelectasis (13). Then, a course of diuretics (hydrochlorothiazide and spironolactone) was commenced. The infant was not switched to non-invasive respiratory support, given the normal work of breathing, oxygen was administered to maintain saturation levels above 95% (14). The respiratory status rapidly improved and six days later (37 + 6 weeks of PMA), the patient no longer needed supplemental oxygen (Figure 3). TnEcho and NT-proBNP improved in two days and normalized in seven days. The LUS also improved in 2 days with resolution of the posterior atelectasis. LUS was periodically performed and excluded the recurrence of atelectasis. The patient was diagnosed with grade I BPD (12) complicated by PH, successfully treated with lung expansion optimization and diuretics (successfully stopped at 41 weeks of PMA), with no need for vasodilator therapies. The baby was discharged home in good conditions.

## Discussion

POCUS has gained a central role in neonatal intensive care over the last years, as a valuable adjunct to clinical assessment (15, 16). Cardiopulmonary ultrasound (CPUS), the combination of LUS and functional echocardiography, has been shown to improve diagnostic accuracy for the identification of underlying etiology in adult patients with acute respiratory failure, frequently guiding clinical diagnosis and/or treatment (17, 18). The use of CPUS in the NICU is still largely unexplored. In this report of four cases, we provide some examples of how CPUS may offer crucial physiopathological information in sick preterm infants (4).

BPD can cause chronic respiratory failure through different mechanisms: arrest of lung and pulmonary vascular development, bronchial hyper-reactivity, bronchial clearance disfunction leading to recurrent atelectasis, diaphragmatic disfunction, large airways disease, spontaneous rib fractures due to osteopenia of prematurity (4, 19–21). Therefore, BPD, defined as oxygen dependency at 36 weeks PMA, is now considered as a common symptom to different disease phenotypes. The gold standard tools to reach a more precise phenotype interpretation (computed tomography, cardiac catheterization, bronchoscopy) are either invasive, require radiation exposure, or are not accessible to most centers (19). An integrated POCUS assessment, including TnEcho and LUS, combined with biomarkers, such as NT-proBNP, may constitute a safe and accessible approach to classify respiratory patterns and longitudinally assess preterm infants with chronic respiratory symptoms.

Among BPD phenotypes, interstitial disorders are a possible, target for specific available treatments. An increase in fibrotic tissue and widening of the interstitial spaces are frequently observed in ex-preterm infants suffering from BPD and undergoing lung biopsy during childhood. Infants with BPD are also prone to an increase in pulmonary interstitial fluid, presumably due to inadequate pulmonary lymphatic drainage. Pulmonary edema may be exacerbated by the presence of a PDA or by a capillary leak secondary to inflammation (22).

Studies enrolling adult patients prove that LUS may be a valuable aid in providing differential diagnoses for interstitial diseases (23, 24). In particular, B-lines due to pulmonary fibrosis/inflammation generally start at the posterior lung basis and are often associated with irregularities of the pleural line and subpleural small consolidations (25). In this pattern of lung injury, the distribution of artifacts is inhomogeneous: spared areas of normal sonographic lung appearance are usually surrounded by areas containing multiple B-lines and pleural line irregularities. In contrast, B-lines are usually detected in a more homogenous, gravity-related distribution, in cardiogenic pulmonary edema (25–27).

In adults suffering from interstitial lung disease, the use of high doses of steroids is proven to significantly improve survival (28), while diuretics are the widely-used symptomatic treatment for pulmonary edema.

In our report, we borrowed these concepts from adult medicine, employing LUS to distinguish the different interstitial phenotypes of BPD, showing a consistent response to targeted treatment. Patients 1 and 3 were classified as congestive LUS pattern, given the regular gravity-dependent distribution of B-lines. This was confirmed by repeating LUS examination after the position change. The use of TnEcho allowed us to differentiate the etiology of pulmonary edema. Differently from Patients 1 and 3, LUS showed multiple diffuse B-lines with irregular distribution non-gravity-dependent in both the anterior and posterior lung fields of Patient 2. Furthermore, the pleural line appeared thickened, coarse, and irregular. Taken together, these findings suggested an interstitial disorder related to fibrosis/inflammation rather than pulmonary congestion.

The use of diuretics or late steroids to prevent BPD is proven ineffective (26, 27). However, the possibility to select those infants that show sonographic features of pulmonary edema vs. signs of ILD may allow targeting patients who could benefit from steroids or diuretics at an early stage of the disease. Moreover, LUS may guide the best timing for treatment and longitudinally monitor response to drug therapy.

Patient 3 showed sonographic features of pulmonary edema secondary to PDA. PDA is associated with several complications of prematurity including BPD, but there is a lack of evidence supporting interventions to close PDA in the attempt to improve respiratory outcomes (29–31). Recently, bedside CPUS has been reported as a good indicator of lung content in neonates with PDA, predicting the possibility of hemodynamic changes in persistent PDA (32). However, LUS is not yet included in the algorithm of PDA management. In patient 3 CPUS evaluation supported the choice to treat PDA, as the entity of pulmonary overflow had led to pulmonary congestion and atelectasis of the dependent regions. Considering the importance of the optimization of patient positioning in adults suffering from pulmonary edema and respiratory failure (33), we used LUS to guide the patient position and mitigate lung de-recruitment of the dependent regions until PDA surgical closure.

Although it is possible that patient 4 suffered from food aspiration with reactive transient pulmonary hypertension, his CPUS pattern was interpreted as a late PH associated with BPD (BPD-PH), as the patient had been symptomatic for two days before CPUS evaluation and had no evidence of reflux or vomiting. BPD-PH is the most recognized BPD phenotype in clinical practice (4). The patient was treated with diuretics as per European guidelines (34), aiming to increase re-absorption of the interstitial lung fluid, potentially improving lung compliance and decreasing pulmonary vascular resistance. Given the good response to this first therapeutic, step, there was no need to escalate therapy towards selective vasodilators. A comprehensive CPUS assessment, showed localized lung atelectasis and allowed to guide the patient's positioning, to recruit the collapsed respiratory units and optimize pulmonary hemodynamics (35). NT-proBNP was used to monitor therapy response (14, 36). Interestingly, the NT-proBNP trend was consistent with the initial LUS findings and with the changes after treatment, highlighting that the management of these patients may successfully include multiple diagnostic/prognostic tools.

A growing body of literature supports the contribution of antenatal factors to the pathogenesis of BPD (37, 38). The multifactorial origin of BPD seems to be related to a combination of different endotypes of prematurity with distinct pathophysiological mechanisms (39). Moreover, a sub-optimal prenatal and early postnatal environment increases the risk of long-term development of adult chronic diseases (36). In the reported cases, the histological examination of the placental material and the identification of the endotypes of prematurity were consistent with the CPUS pattern. Patient 2, who was diagnosed with ILD at LUS, had an infective/inflammatory histological endotype of prematurity. Patient 4 presented the more established association between the vascular phenotype of BPD and the endotype of dysfunctional placentation (37). In patients with a congestive pattern, the placental examination was unremarkable. Further research is warranted to investigate the correlation between phenotypes of BPD classified with POCUS and endotypes of prematurity assessed with placental histology.

Our experience suggests that POCUS may offer a targeted approach in terms of type and timing of intervention, in order to optimize treatment success and avoid exposure to unnecessary or untimely therapies. The identification of specific patterns at CPUS evaluation and their association with the underlying physiopathology of the endotypes of prematurity may be crucial in the investigation of therapies for preterm infants requiring protracted respiratory support.

Our findings need to be confirmed by large randomized clinical trials, before entering daily clinical practice.

## Data Availability

The original contributions presented in the study are included in the article/Supplementary Materials, further inquiries can be directed to the corresponding author.
